# ‘Lessons learned’ from trialists who adapted a complex intervention for remote delivery within a trial as a result of the COVID-19 pandemic: a scoping review

**DOI:** 10.1186/s13063-025-09145-6

**Published:** 2025-11-25

**Authors:** Ella Howes, Samuel G. Smith, Katie Gillies, Lisa Zhang, Amanda J. Farrin

**Affiliations:** 1 Leeds Institute of Clinical Trials Research, University of Leeds, Leeds, UK; 2 Leeds Institute of Health Sciences, University of Leeds, Leeds, UK; 3 Aberdeen Centre for Evaluation, University of Aberdeen, Aberdeen, UK; 4 Department of Psychology, University of Bath, Bath, UK; 5 Edinburgh Clinical Trials Unit, University of Edinburgh, Edinburgh, UK

## Abstract

**Background:**

During the COVID-19 pandemic, complex interventions being evaluated in randomised controlled trials were often rapidly adapted from in-person to remote delivery. Such adaptations to intervention delivery have the potential to cause unintended consequences and affect important aspects of trial generalisability and interpretation. This scoping review aimed to identify the ‘lessons learned’ from trialists who adapted and remotely delivered a complex intervention within a trial because of the COVID-19 pandemic. Gaining a better understanding of trialists’ experiences of adapting interventions for remote delivery will identify where more in-depth investigation and guidance is needed.

**Methods:**

The Joanna Briggs Institute (JBI) scoping review guidelines were followed. The search was developed for MEDLINE and adapted for Web of Science, PsycINFO, EMBASE, and Cochrane. Data were extracted on study characteristics, methods reported to adapt interventions, and the challenges and facilitators of the process of adaptation and remote intervention delivery. Data on remote intervention delivery were organised using the upper level of the Behaviour Change Intervention Ontology.

**Results:**

Fifteen articles were eligible for inclusion describing insights from 16 randomised controlled trials, across a range of populations and trial designs. Most discussion focused on challenges and facilitators of the remote delivery of the complex intervention. These included privacy and safety concerns of intervention delivery within the home setting, and technological issues of remote delivery via video call. The most frequently reported facilitator was the use of an environmental inventory before intervention delivery to check the space in which participants were located, and the materials available to them.

**Conclusion:**

Suitability of an intervention for remote delivery depends not only on whether it is originally delivered via a digital technology, but also the extent to which it requires human facilitation and support. Privacy and safety concerns in the home environment could impact trial participation in a remotely delivered intervention. Further research is needed to explore how trialists can effectively prepare for and manage the challenges of remote intervention delivery. Guidance developed to support adaptation of an intervention for remote delivery within a trial should be specific to the mode of delivery used.

**Supplementary Information:**

The online version contains supplementary material available at 10.1186/s13063-025-09145-6.

## Introduction

In March 2020, many trialists evaluating complex interventions faced an unprecedented adaptation situation. For a trial to continue during the COVID-19 pandemic restrictions, an intervention had to be rapidly adapted from face-to-face to remote delivery, rather than being designed to be delivered remotely from the outset. Decisions about how best to adapt a complex intervention, however, are not straightforward [1]. A single adaptation can cause a number of unintended consequences and affect important aspects of trial generalisability and interpretation [2, 3]. Without clear signposting and guidance, trialists will have responded to these challenges in the best way they could [4]. There is much we can learn from this period about what trialists need to consider when adapting a complex intervention for remote delivery in the future.

Complex interventions are typically defined as interventions with multiple different, interacting components [5, 6]. One of these interacting components is intervention mode of delivery, which can be defined as the method or methods by which intervention content is brought to its target population [7], for example, via video call or telephone [8]. During the COVID-19 pandemic, trialists had little opportunity to consider the appropriateness of intervention mode of delivery with the study design and population. Decisions were often guided by the ability to deliver intervention content remotely within pandemic lockdown restrictions, typically achieved using digital technologies [9]. However, intervention content does not always translate seamlessly from one mode of delivery to another [10]. Additional adaptations would likely have been required that could mean the intervention adapted for remote delivery is not the equivalent to that delivered before adaptation [2]. Furthermore, trialists will have had to consider how participants could engage with the intervention in their own homes, instead of the originally planned setting. This likely required additional planning and logistics, particularly if delivering the intervention via digital technologies [11, 12].

While guidance exists to support the adaptation of complex interventions [13–16], considerations for the adaptation of mode of delivery during the evaluation stages are lacking. Furthermore, the adaptation of complex interventions for remote delivery via digital technologies requires specific considerations of the environment in which the intervention is being implemented [14, 17]. There is limited evidence on how best to implement interventions remotely using digital technologies within a trial [18].

A scoping review can provide an overview of a broad research field and identify areas for more in-depth research [19]. This is particularly valuable where evidence is widely dispersed and extensive [20], as is the case for research from the COVID-19 period. Gaining a better understanding of trialists’ experiences of adapting interventions for remote delivery during the COVID-19 pandemic will identify where more in-depth investigation and guidance is needed to support remote intervention delivery within a trial. This learning is particularly relevant for the conduct of future trials within health and care services that have changed to more remote delivery models as a result of COVID-19 [21].

### Aims and objectives

This scoping review aims to explore the literature reporting interventions that were adapted to remote delivery within a trial as a result of the COVID-19 pandemic to identify the ‘lessons learned’ related to intervention *adaptation* and *remote delivery*. Specifically, it aims to 1. identify the methods trial teams reported to adapt a complex intervention for remote delivery and 2. identify the challenges and facilitators of the process of adaptation of a complex intervention and its remote delivery.

### Methods

The review was conducted and is reported according to the JBI methodology for scoping reviews which provides a structured approach to defining objectives, identifying relevant studies, and synthesising evidence [20]. It is reported in line with the Preferred Reporting Items for Systematic Reviews and Meta-Analyses extension for scoping reviews (PRISMA-ScR) guidelines [22]. The review followed an a priori protocol, which is registered and publicly available on the Open Science Framework [23].

### Search strategy

An initial limited search of PUBMED and SCOPUS was undertaken in May 2024 to identify articles on the review topic and inform the search strategy. In accordance with the JBI manual, a selection of articles was used to identify keywords and phrases related to this study [24–31]. The final search terms incorporated a combination of four key concepts from the research question: 1. adaptation, 2. complex intervention, 3. remote delivery, and 4. randomised controlled trial. An information specialist was consulted to develop the final search strategy. The original search was developed for MEDLINE and adapted for Web of Science, PsycINFO, EMBASE, and COCHRANE.

The term ‘COVID-19’ was not used in the search due to the large number of papers retrieved. Searches were restricted to March 2020–May 2024, with May 2024 reflecting the date the searches were conducted. This timeframe ensured the inclusion of literature published during the COVID-19 period. Although the WHO declared COVID-19 was no longer a global emergency in May 2023, relevant literature continues to be published beyond this point. There was a lack of consistency in how papers related to ‘lessons learned’ had been indexed. Therefore, MESH terms were not used. The full search strategy is presented in Additional file 1.

### Eligibility (inclusion/exclusion) criteria

This scoping review considered studies reporting quantitative, qualitative, and mixed methods research. Reviews in any form (literature, scoping, or systematic) that met the criteria were considered. Primary studies included in existing reviews were assessed for eligibility. Conference abstracts and protocols were not considered.

This review used the Participant, Concept, Context (PCC) approach for developing eligibility criteria [20]. We included research literature that met the following PCC criteria in this scoping review.

### Population

The focus of our scoping review is lessons learned from trialists delivering trials of complex interventions conducted on human participants.

### Concept

The key concepts are ‘lessons learned’ about adapting a complex intervention for remote delivery within a trial as a result of the COVID-19 pandemic. This is further specified by [1] methods taken by trial teams to adapt a complex intervention for remote delivery; [2] challenges and facilitators related to the process of adapting a complex intervention to remote delivery within a trial; and [3] challenges and facilitators related to the remote delivery of a complex intervention within a trial.

Moore et al. [16] define adaptation as the ‘intentional modification(s) of an evidence-informed intervention, in order to achieve a better fit between an intervention and a new context’. They distinguish between planned adaptations and responsive adaptations. This scoping review is interested in understanding the challenges and facilitators of the responsive adaptations that trial teams made to a complex intervention as a result of the COVID-19 pandemic.

The NIHR Remote Trial Delivery Working Group defines remote delivery as ‘all activities related to clinical trial delivery undertaken without in-person, face-to-face contact’ [4]. This review is interested in the challenges and facilitators of remote delivery of the intervention being evaluated (as opposed to other trial processes such as recruitment and collecting consent).

Methods taken to adapt a complex intervention are defined as any process a trial team takes to adapt an intervention from in-person to remote delivery.

### Context

Reports were included from randomised controlled trials (pilot/feasibility/phase 3 or 4) conducted in any geographical location. Reports published between March 2020 and May 2024 were included in this review. This review team selected March 2020 as the cut-off as this was when the World Health Organisation declared COVID-19 a pandemic.

### Identification of eligible studies

Following the search, all identified citations were uploaded into Rayyan [32] and duplicates were removed. Titles and abstracts were screened by EH against the eligibility criteria for the review, and a random 10% were checked by LZ. Potentially relevant reports that met the PCC criteria outlined above were retrieved in full and their details imported into Rayyan. The full text of these reports was assessed in detail against the eligibility criteria by EH with a further 20% checked by LZ. Discrepancies were discussed and resolved by EH and LZ. Reasons for exclusion at the full text stage were recorded.

### Data extraction

Data were extracted from papers included in the scoping review by EH using a data extraction tool developed for the purpose of this review. The tool was used to extract specific details about the publication as well as data related to the review questions. Extracted data on study characteristics included country of origin, study design, and aims. The data extracted for the review questions included mode of delivery to deliver intervention content remotely, methods reported to adapt the intervention, and challenges and facilitators of the process of adaptation and of remote intervention delivery. We deviated from the protocol by not extracting data on ‘adaptations made to the intervention’ and ‘adaptations made to comparator’ as data was not easily presentable in the studies included. See Additional file 2 for the data extraction tool.

The table was piloted by EH using a sample of three full-text articles. Issues were discussed with AJF, SS, and KG, and the extraction table modified and revised accordingly.

### Data analysis and presentation

We used the upper level of the Behaviour Change Intervention Ontology (BCIO) [33] as a framework to organise the challenges and facilitators related to remote intervention delivery.

**Fig. 1:**
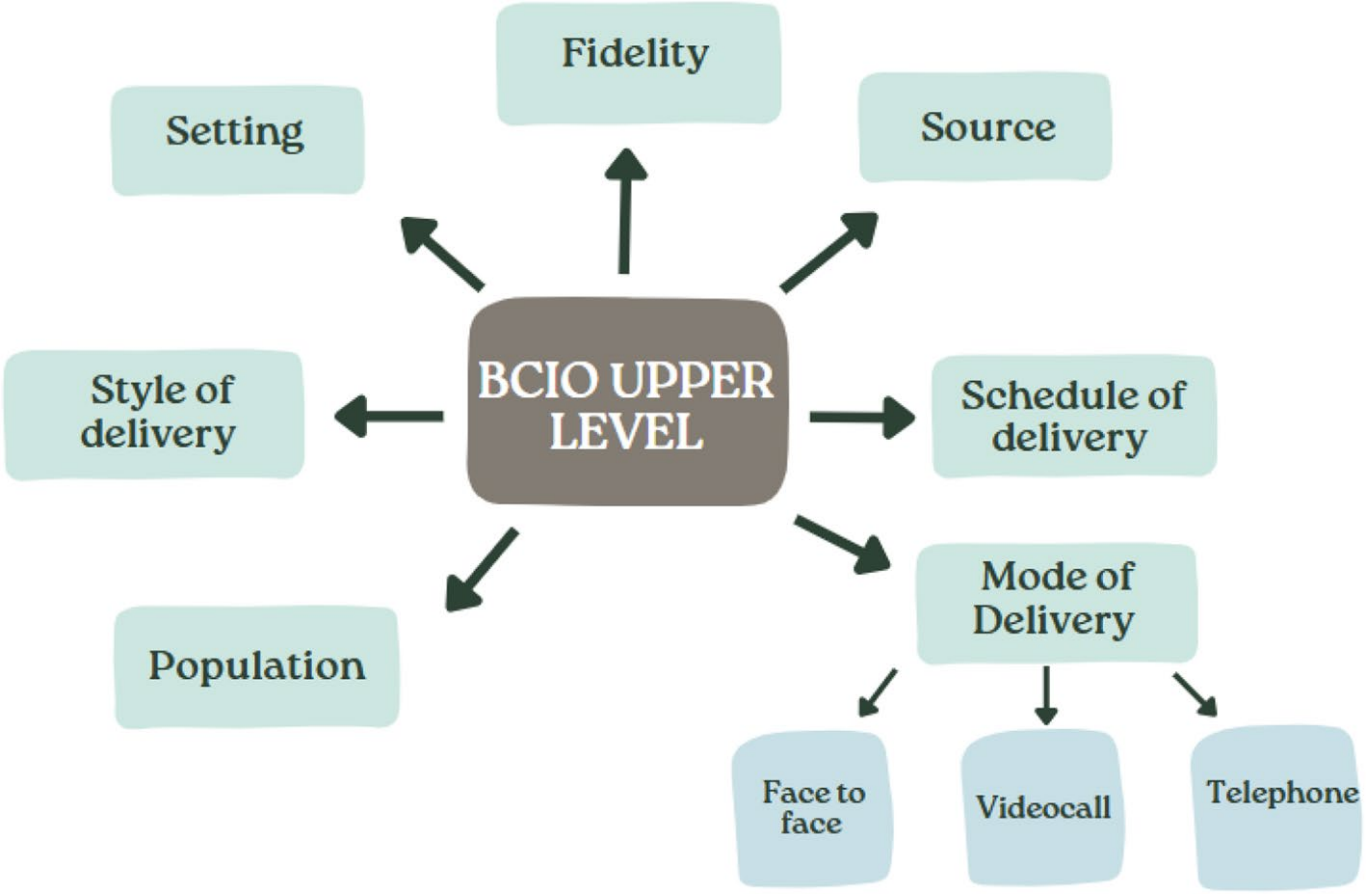
Upper level of the Behaviour Change Intervention Ontology adapted from Michie et al. [33] with example lower classes of mode of delivery

An ontology represents knowledge in a domain by defining key entities within it and the relationship between them [34]. The BCIO upper level provides a high-level classification of the components of a behaviour change intervention and its evaluation [35]. The BCIO has applicability to complex interventions more generally. Firstly, the majority of complex interventions have a behavioural aspect [36]. Secondly, the development of the BCIO was informed by the CONSORT guidelines for reporting of clinical trials and the Template for Intervention Description and Replication (TIDieR) [37–39]. These frameworks are used to support the reporting of complex interventions in trials (Fig. 1).

Each challenge or facilitator that related to the remote delivery of the complex intervention was mapped according to the BCIO upper level. Data within each category was grouped and descriptively summarised [40]. This supported a more granular understanding of the challenges and facilitators that related to remote delivery. For example, data was organised depending on whether a specific challenge related more to the mode in which the intervention was delivered (e.g. via telephone or video call), or the setting in which it was remotely delivered. It also provided an organised and effective way to present the large quantity of qualitative data. A second reviewer (LZ) cross-checked the grouping of the data. Disagreements were resolved through discussion.

Study characteristics were summarised using descriptive statistics. The challenges and facilitators of adaptation and remote intervention delivery were presented in a table and aligned where possible. The challenges and facilitators of adaptation and remote intervention delivery were also summarised narratively. No quality assessment of the included articles was conducted since the aim of the scoping review was to map the breadth of emerging evidence regarding trialists’ experience of adapting a complex intervention for remote delivery, not to critique the quality of included articles.

## Results

### Identified studies

**Fig. 2:**
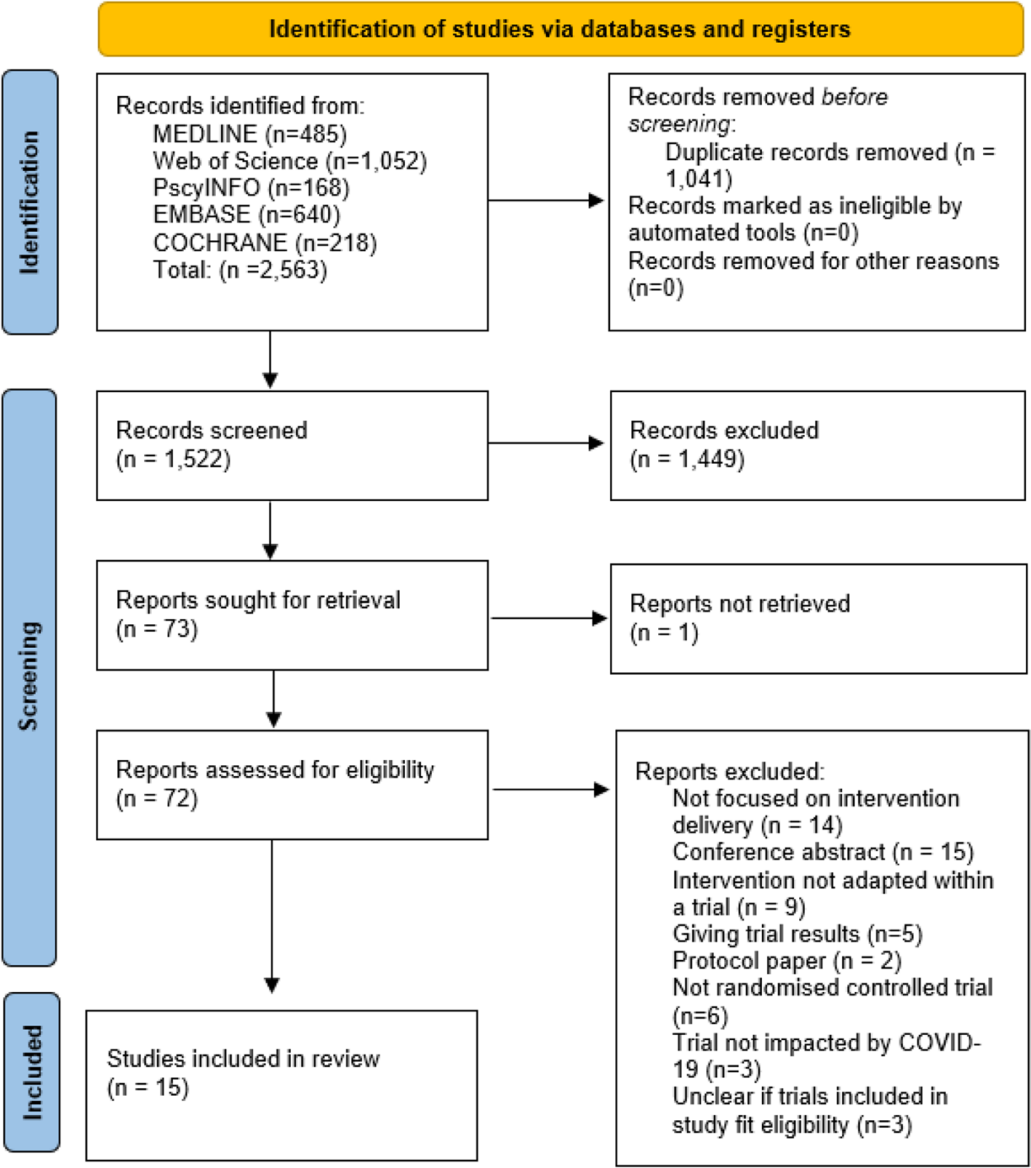
PRISMA flow diagram

In the initial database search, 2563 records were identified. Once duplicates were removed, 1522 titles and abstracts were screened for inclusion. Seventy-three full-text studies were assessed for eligibility. Fifteen papers met the eligibility criteria. The search process and results are documented in the PRISMA flow diagram. One paper could not be found online. The authors were contacted but the full text was not retrieved (Fig. 2).

### Characteristics of included studies

Fifteen studies were included, describing insights from 16 randomised controlled trials. Of the 15 reports identified, 13 were from the USA, 1 from Canada, and 1 from the UK. Eleven of the 16 trials were two-arm trials [24, 28, 41–49], three were three-arm trials [50–52], and two were four-arm trials [31, 53]. Most trials were evaluating an intervention that involved a psychotherapy component, such as motivational interviewing and cognitive behavioural therapy (*n* = 8) and/or an exercise component (*n* = 6). Three of the trials included were group-based interventions [31, 43, 48]. A range of modes of delivery were used to remotely deliver the intervention. The most common mode of delivery used for remote delivery was video call (*n* = 15). As demonstrated in Table 1, there was a range of participants included in the trials included in this review.

**Table 1:** Study characteristics of included studies

Authors and date	Country	Included study aim	Randomised controlled trial design	Target population and planned sample size	Intervention And Comparator	Mode(s) of delivery of originally planned intervention							Mode(s) of delivery of intervention adapted for remote delivery						
						Face to face	Video call	Telephone	Paper materials*	Text messages	Computerised**	Not clear	Face to face	Video call	Telephone	Paper materials*	Text messages	Computerised**	Not clear
Binder 2021 [50]	USA	Report of the methods of this study, including adjustments made to the protocol because of the coronavirus-2019 disease (COVID-19) pandemic	A three-arm, individually randomised, phase 3 trial of an exercise and testosterone therapy	Women > 65 with a recent hip fracture *n* = 300	Supervised exercise + testosterone gel***	X									X	X			
					supervised exercise and placebo gel	X								X	X				
					Enhanced usual care (home exercise + health education (Comparator)	X									X		X		
Everhart 2021 [53]	USA	Report to provide updates on modifications made to the RVA Breaths Trial	A four-arm, cluster randomised, phase 3 trial of an asthma education intervention (RVA breaths)	Children (aged 5–11) in low-income areas *n* = 300	Asthma education with community health workers	X		X						X	X	X			
					Home remediation with home assessors, and a school nurse component	X			X					X		X			
					Asthma education and home remediation	X								X	X				
					Enhanced standard of care (comparator)				X							X			
Fanning 2023 [31]	USA	Report to describe process of adapting the IMOVE intervention for delivery via videocall in response to COVID-19	A 2X2 randomised factorial pilot trial of a group-based, improvisational dance intervention	Adults with early-stage Alzheimer’s Disease and their caregiver *n* = 80 (40 dyads)	Movement group (movement and social)	X								X					
					Movement alone	X								X					
					Social Group	X								X					
					Usual Group							X		X					
Fisher 2021 [41]	USA	Report to outline the procedural modifications used to facilitate conversion of an in-person intervention to remote delivery	A two-arm individually randomised pilot trial of a cognitive behavioural therapy (CBT) for depression	Individuals aged 21–69 with traumatic brain injury *n* = 40	Cognitive Behavioural Therapy	X					X			X	X	X			
					Control: 12-week waitlist	-	-	-	-	-	-	-	-	-	-	-	-	-	-
Johnson 2022 [42]	USA	Report to describe study challenges and adaptations as a result of COVID-19 research restrictions	A two-arm individually randomised phase 3 trial of a healthy relationship tool and safety decision aid to reduce intimate partner violence (IPV) during perinatal period	Women aged 18–45 who screen positive for IPV *n* = 186	Online healthy relationship tool and safety decision aid	X					X				X			X	
					Attention-time information matched control condition							X							X
Kashikar-Zuck 2022 [51]	USA	Report to describe modifications made to the FIT Teens study protocol in response to the COVID-19 pandemic	A three-arm individually randomised phase 3 multi-site trial of a specialised neuromuscular training intervention with Cognitive Behavioural Therapy	Adolescents aged 12–17 juvenile fibromyalgia *n* = 420	FIT teen intervention (combines Cognitive Behavioural Therapy with neuromuscular training)	X								X		X		X	
					Cognitive Behavioural Therapy only	X			X					X		X		X	
					Graded Aerobic Exercise only	X								X		X		X	
Klasko-Foster 2022 [29]	USA	Report to provide descriptions of remote adaptations to two RCTs of behavioural interventions for primary and secondary HIV prevention****	1) A two-arm individually randomised phase 3 trial to establish the efficacy of a behavioural intervention to optimize ART adherence and viral suppression [44]	HIV-infected youth, ages 16 to 29 *n* = 192	Text messaging reminders to take medication and in-person counselling	X				X				X					
					Standard of care							X							X
			2) A three-arm individually randomised phase 3 trial testing an integrated HIV risk reduction and behavioural activation counselling intervention (IMPACT) for HIV-uninfected, stimulant [52]	Men aged 18 or older *n* = 286	Behavioural Activation and Cognitive Behavioural Therapy with HIV sexual risk reduction counselling	X								X					
					A time- and intensity-matched control including relaxation therapy and educational support with counselling	X								X					
					A standard of care (SOC) comparison condition including counselling only	X								X					
Lalande 2021 [28]	USA	A tutorial to help clinical researchers transition their in-person research programs to a web-based format	A two-arm phase 3 trial, individually randomised at the dyad, of a relationship-education counselling program for couples	Couples in which one partner has experienced a cardiac event *n* = 608	Healing Hearts Together counselling programme	X								X		X			
					Usual care at the hospital							X							X
McCourt 2022 [43]	UK	A report to document changes to a previously published protocol, as well as facilitate study design in the immediate post pandemic era	A two-arm, individually randomised, pilot trial of an exercise prehabilitation before and during stem cell transplantation	Patients with multiple myeloma *n* = 60–75	Group-based supervised exercise	X		X						X					
					Usual care							X							X
Meline 2021 [24]	USA	A report to describe the experience of transitioning to virtual methods to deliver intervention and conduct study visits	A two-arm, individually randomised, phase 3 trial of a lung cancer screening decision tool	Veterans aged 55–80 with a history of smoking *n* = 200	A lung Cancer Screening Decision Tool (LCSDecTool) website	X			X		X				X			X	
					Control intervention website	X					X								X
Shear 2022 [45]	USA	A report to provide lessons learned throughout the process of adapting to a virtual format	A two-arm, individually randomised phase 3 trial examining graded exposure treatment compared to multidisciplinary pain management	Youth aged 8–18 with Chronic muscular-skeletal pain *n* = 74	Graded Exposure Treatment	X			X					X		X			
					Multidisciplinary Pain Management (outpatient Cognitive Behavioural Therapy and physical therapy)	X								X					X
Sperl-Hillen 2021 [46]	USA	Report to describe an RCT to improve chronic kidney disease (CKD) care and the challenges encountered due to COVID-19	A two-arm cluster randomised pragmatic trial to test a chronic kidney disease clinical decision support tool	Patients with stage 3 or 4 chronic kidney disease n = 47 primary care clinics	Chronic-kidney disease clinical decision support (CKD-CDS)	X					X			X	X			X	
					Usual care							X							X
Tang 2021 [47]	Canada	A report to examine health educators’ experiences and perceptions of transitioning from home visits to remote delivery of motivational interviewing	A two-arm cluster randomised, phase 3 trial to test the impact of a home-based obesity prevention intervention	Families with children between ages 1.5–5 *n* = 285	Motivational interviewing	X				X				X	X				
					Control							X							X
Vinci 2022 [48]	USA	A report to describe the technical and logistical considerations of transitioning from in-person to remote-based delivery for group-based treatment	A two-arm group-based individually randomised pilot trial for smoking cessation/alcohol used	Adults aged 18 + who are daily cigarette smokers *n* = 64	Mindfulness-Based Relapse Prevention for Smoking and Alcohol (MBRP-SA)	X								X	X				
					Cognitive Behavioural Therapy	X								X	X				
Zaccari 2022 [49]	USA	A report to present modifications made to pivot a multisite RCT from in-person to virtual study implementation	A two-arm group-based individually randomised pilot trial of a yoga and cognitive processing group therapy	Women veterans with Post-Traumatic Stress Disorder (PTSD) *n* = 40	Cognitive Processing Therapy (CPT)	X			X					X		X			
					Trauma Centre Trauma Sensitive Yoga (TCTSY)	X								X		X			

^*^Printed materials were given as a hard copy to participants in the original intervention. In the adapted intervention, these materials were either posted and/or emailed to participants to print

^**^Computerised mode(s) of delivery captures intervention content that was viewed/interacted with via a computer (e.g. on a website or video)

^***^ Women were told to continue their prescribed gel (provided to them before the pandemic) unless it was determined it was unsafe

^****^ Trial description has come from the protocol paper for the two papers discussed in this study

### Findings

Most papers reported that they had adapted the intervention in order for it to be conducted remotely, but provided little detail about how this was achieved [24, 29, 41, 42, 45–51]. Five studies briefly reflected on the challenges and facilitators of adaptation [24, 28, 31, 45, 53]. The majority of discussion reported focused on the challenges and facilitators of the remote delivery of the complex intervention. Most of these challenges related to the setting in which an intervention was delivered (e.g. challenges around privacy and safety), and the mode of delivery in which an intervention was delivered (e.g. challenges of remote delivery via video call).

### Methods that trialists report using to adapt a complex intervention for remote delivery

Four papers highlight that participants were involved in the adaptation process [28, 31, 43, 53], for example, by consulting them on the proposed changes and gaining their approval [43]. One study of the adaptation of a group-based movement intervention highlighted how they aimed for fidelity to key aspects that were hypothesised to be important for improving quality of life [31]. A second study, which was written as a tutorial for researchers adapting to web-based formats, detailed five steps undertaken [28]. This included consultation and assessments of needs, adaptation of procedures for remote delivery, adaptation of procedures and materials for the research protocol, pilot sessions, and the final launch. Feedback was gathered in the pilot stages from participants who received the in-person intervention. The authors specified how this feedback contributed to treatment fidelity and the acceptability of the web-based format. Lalande et al. [28] report that the iterative process they took to adapt the intervention was guided by a four-step framework outlined by Hom et al. [54]. This included consultation, adaptation, pilot testing, and test launch.

### Challenges and facilitators of the process of adapting a complex intervention for remote delivery

Five studies briefly reflected on the challenges and facilitators of adaptation [24, 28, 31, 45, 53]. Challenges discussed included the time available to iteratively adapt the intervention and specific features of the intervention that made it more difficult to adapt. For example, it was more challenging to adapt the intervention when there were more interactive features [24]. Key facilitators reported included consulting with others and good communication among members of the team [24, 28] (Table 2).

**Table 2:** Challenges and facilitators of adaptation

Challenges	Facilitators
• The more complex the intervention, the more refinement it needed [31]	• Good communication among members of the research team facilitated the adaptation process [24, 28]
• Limited time available to iteratively adapt and get feedback on acceptability and feasibility [45]	
• Specific features of an intervention, such as the amount of interactive features [24] and the degree to which the intervention component was non-verbal [31]	• Having team members with expertise in the programming of the intervention tools [24]
	• Consulting with others, such as trial participants and members of the team [28, 53]

### Challenges and facilitators of remote intervention delivery

All reports discussed challenges and facilitators related to remote intervention delivery. These challenges and facilitators have been organised using the upper level of the Behaviour Change Intervention Ontology [33]. Specifically, they are organised according to whether the challenges and facilitators are mostly related to the setting in which the intervention was delivered (setting), the individual delivering the intervention (source), the mode in which intervention content was delivered (mode of delivery), the style in which an intervention was communicated (style of delivery), how often the intervention could be delivered (schedule of delivery), the way in which remote delivery of the intervention impacted intervention fidelity (fidelity), and any participant characteristics that made remote intervention delivery more or less challenging (population). Each of these is discussed in detail below. Table 3 details the challenges and facilitators identified within each of these categories.

**Table 3:** Challenges and facilitators of remote intervention delivery

BCIO upper level	Challenges	Potential facilitators
**Setting** *The environment in which an intervention is delivered* [79]	• Privacy to engage in the intervention in the home environment [29, 48, 49, 51]	• Carrying out an environmental inventory/checklist to identify if participants were in a private space [48] • Additional precautions implemented (e.g. at the start of group sessions participants are reminded that they must be in a private space or else they must leave the session) [51]
	• Availability of space in the home [45]	• Carrying out an environmental inventory/checklist to identify if participant had movement space of a sufficient size [31]
	• Equipment availability [45, 50, 51]	• Carrying out an environmental inventory/checklist to identify if participants had the equipment needed to carry out the intervention in their home [28, 31, 45, 48, 53]
	• Interruptions and distractions in the home environment [45, 48, 49]	• Providing extra information and instruction on how to limit distraction during the session [41]
	• Safety concerns related to the risk of falling from completing certain exercises [43, 45, 50] • Safety issues related to who participants live with [46]	• Identifying participants physical location before the start of intervention delivery [41] • Remove certain balance exercise from intervention [50] • Updating eligibility criteria for vulnerable participants [42] and those at risk of falling [43]
	• Exercising in non-air-conditioned homes in hot weather [45]	
**Source** *The person, population, or organisation that delivers the intervention* [80]	• Dealing with distractions and interruptions [31, 48]	
	• Difficulties building rapport with participants [47, 48]	• Providing additional training on how to build rapport online [53]
	• Difficulties as a result of not being ‘in the room’ [24, 28, 45]	
	• Extra burden placed on the intervention source [24, 47, 48, 51] • Emotional fatigue experienced by staff as a result of participants turning to them for support during the COVID-19 pandemic [51]	• Providing extra support for the interventions source [29]
	• Questioning importance of role in a virtual setting [53]	• Making deliberate efforts to validate contributions of individuals delivering the intervention [53]
		• Providing additional training around using videoconferencing software [28, 48]
**Style of delivery** *The manner in which intervention content is communicated to intervention participants* [81]	• Inability to read subtle non-verbal cues and eye contact [31, 48]	• Increasing quantity of communication with participants [29, 46, 53] • Adding extra time specifically to build rapport and increase bonding [31, 48, 53]
	• Reliance on verbal instruction and cues [45] as therapists were unable to make corrections to form as they might normally do	• Describing what should be felt during an exercise [45] • Using agenda-setting to let participants know what will be happening and using plain language to describe new procedures [29]
	• Risk of conversation being dominated by one member of the group [31]	• Active management of group dynamics [31]
		• Using open-ended questions to understand participant experiences of COVID-19 [45]
**Fidelity** *The degree to which an intervention is put in practice as intended* [61]		• Updating training for the intervention source [28, 51], for example, by creating training videos around the updated study protocols [51]
	• Challenges with fidelity assessment [24, 46, 48, 49]. For example, issues related to being unable to directly observe participants and record their progression [24] and confidentiality issues around recording intervention delivery online [48, 49]	
	• Uncertainty of intervention receipt [24, 45, 46]. For example, uncertainty if materials had been printed and/or received in the mail [24, 45]	
	• Missed/forgotten sessions online [45]	• Providing text and email reminders for attendance to the virtual settings [45]
	• Internet issues accessing an online tool [24]	
**Mode of delivery** *The method or methods by which intervention content is brought to its target population* [7]	**Video call (e.g. Zoom, Microsoft Teams)**	
	• Technological issues related to video conferencing software [24, 29, 31, 45, 47, 49, 51] which one paper reported as being heightened in a group setting [49]	• Providing extra instruction, technological support and assistance for participants [28, 31, 41, 48, 49] • Spending time prior to intervention delivery to check equipment was functioning and participants were comfortable with the set up [28, 31, 41] • Having a plan in place for navigating potential difficulties [29, 51, 53] for example, a back-up plan for what participants should do if connection is lost [51] and agreeing on a cut-off point at which the intervention was delivered via phone instead of via video conferencing
	• Risk of spyware for a vulnerable participant group [42]	
	• Difficulties from using devices with different sized screens to engage in the intervention. For example, using a phone or an iPad [48]	
	• Access to devices in the home if delivering an intervention to more than one person at the same time (e.g. a parent and a child) [45]	• Providing technological equipment for participants [41, 48], for example, loaning tablets for those that needed
	**Audio call (telephone)**	
	• Participants more distracted on the phone [47]	
	• Non-verbal cues not possible [47]	• Being ‘more comfortable with the silence’ [47] • Asking more probing questions [47]
	**Printed material (posted or emailed to participants)**	
	• Difficulties mailing materials to participants with unstable housing [29]	• Having an alternative location available for participants to collect materials, and leveraging community partnerships [29]
	• Uncertainty if intervention materials have been received [24]	
	• Disruptions to mailing service [48]	
	• Sending out materials was resource consuming [48]	
	• Variability in who printed the materials that were sent to them [45]	
	**Computerised (website)**	
	• User experience impacted by screen size [24]	
	• Computer programme being used in the intervention was not designed for screen share [46]	
	• Navigation issues with the intervention might go undetected or misinterpreted [24, 46]	
	• Difficulties accessing the study website as a result of internet access [24]	
**Schedule of delivery** *The duration, frequency, and timing of intervention delivery*	• Missed and forgotten virtual visits [45, 46]	• Providing text and email reminders for attendance to the virtual settings [45]
	• Scheduling difficulties related to changes in the home environment as a result of COVID-19 [47, 50], for example, having children at home while parents worked full time	
		• Increased opportunity to schedule appointments [28, 46, 47]
**Population** *The group of participants involved in the trial*	• Individuals with photosensitivity could not look at the screen for too long [41]	• Reducing the quantity of time participants needed to look at a screen [41]
	• Increased safety risks of engaging with the intervention for vulnerable people who live with abusive partners [42]	• Changing the eligibility criteria of the trial. Specifically, participants were excluded if it was deemed their risk of spyware was too high, or they were at a greater risk for severe injury and/or homicide [42]
	• Safety risks for individuals with balance issues [43]	• Including an extra assessment tool to identify participants with balance issues and make necessary adjustments to their participation [43]

### Setting

All the studies in this review reported that they remotely delivered the adapted intervention into participants’ homes. The key challenge trialists report facing in the home setting related to the privacy of engaging in the intervention [29, 48, 49, 51] and safety concerns related to the risk of falling [43, 45, 50]. Privacy challenges were more commonly associated with interventions that contained a counselling component. Safety issues were more commonly discussed in interventions that contained an exercise component. The most frequently reported facilitator was the use of an environmental inventory/checklist to check the space in which participants were located and the equipment they had available [28, 31, 45, 48, 53].

### Source

Trialists reported challenges the intervention provider faced as a result of not being ‘in the room’ [24, 28, 45]. For example, not being able to physically correct form during exercise [45] or directly observe participants’ progression [24]. Four studies highlighted the extra burden remote delivery placed on the individual delivering the intervention [24, 47, 48, 51]. For example, by increasing the amount of communication needed with participants and the extra time needed to set up a visit. Key facilitators discussed included providing additional training related to building rapport with participants online [53] and videoconferencing skills [28, 48]. In one study, intervention deliverers were provided with more opportunities for debriefing [29].

### Style of delivery

The inability to read non-verbal cues was reported as a challenge to how intervention content could be communicated [31, 48]. Potential solutions included increasing the quantity of communication with participants and adapting communication styles. For example, by using plain language to describe the new procedures [29].

### Fidelity

Remote delivery of the intervention was reported as causing several challenges related to fidelity assessment [24, 46, 48, 49]. These challenges were related to the mode of delivery in which an intervention was delivered. For example, two papers reported uncertainty of intervention receipt when posting printed materials to participants [24, 45]. Recording intervention delivery on a video call to measure fidelity was reported as causing confidentiality issues [48, 49]. Few potential solutions to these challenges were discussed by trialists.

### Mode of delivery

#### Video call

Nearly all papers included in this review reported using video call (e.g. Zoom or Microsoft Teams) to deliver the intervention remotely. This mode of delivery was associated with the greatest number of reported challenges. The majority of these challenges related to technological issues [24, 29, 31, 45, 47, 49, 51]. Potential solutions discussed by trialists mostly related to providing extra instruction, support, and assistance for participants [28, 31, 41, 48, 49]. Two papers discussed how certain issues around relational connectivity and rapport building during a video call were heightened in group-based interventions [28, 53].

#### Audio call (telephone)

Several papers provided the option for intervention delivery via the telephone. One study discussed challenges this caused as a result of the lack of non-verbal cues and the potential for participants to get more distracted [47].

#### Printed materials (posted or emailed to participants)

Several papers that used printed materials to deliver intervention content reported either posting these materials to participants or emailing them to be printed. However, one study highlighted that when materials were emailed, there was variability in which participants printed the materials [45]. They highlight that this could have reduced the saliency of the materials, as participants were less able to write and reflect directly on them.

Posting materials to participants was reported as being a resource-consuming activity [48]. One paper reported challenges if participants had unstable housing [29]. Having an alternative location available to send materials was discussed as a potential facilitator of this [29].

#### Computerised (website)

While the mode of delivery could stay the same for parts of an intervention that were originally delivered via a website, challenges were associated with its remote delivery as a result of not being present with participants while they were set up with the intervention. Other challenges reported included navigation issues going undetected [24, 46] and the website not being designed for screen share on a video call [46]. One study reported that some eligible patients were not able to participate in the intervention because they lacked internet access at home [24].

### Schedule of delivery

Three papers reported the increased opportunity remote delivery provided for scheduling appointments for intervention delivery [28, 46, 47]. For example, it meant there was no need to cancel appointments if a family member was ill or there were parental work commitments [46]. Two papers reported issues with missed and forgotten visits [45, 46]. Providing text reminders was used to address this [45].

### Population

Two papers reported on characteristics of the population for which remote delivery resulted in a few potential safety issues. These included vulnerabilities from a potentially unsafe living situation [42] and safety issues as a result of balance issues [43]. One paper reported changing the eligibility criteria to address this [42]. Specifically, participants were excluded if it was deemed their risk of spyware was too high, or they were at a greater risk for severe injury and/or homicide [42]. An additional balance tool was introduced to identify potential balance issues and adjust participant participation as a result [43].

## Discussion

This scoping review synthesised insights from trialists about the adaptation of a complex intervention to remote delivery as a result of the COVID-19 pandemic. The review identified a broad range of challenges and facilitators of remote delivery of an intervention within a trial. The majority of these challenges related to the setting in which the intervention was delivered, and the mode of delivery used to deliver the intervention remotely. While this review aimed to identify methods reported to adapt interventions, and challenges and facilitators of the adaptation process, few included studies described this. Several areas for which guidance is needed to support trialists to adapt an intervention for remote delivery have been identified.

Most of the challenges identified in this review related to the home setting in which the intervention was remotely delivered. Some of these challenges, such as interruptions in the environment, were likely heightened during the lockdown period when family members were present in the home environment. However, other challenges, such as those related to the privacy and safety of intervention delivery, have been discussed in the pre-pandemic literature [55–58]. A recent review on privacy and confidentiality in telemedicine also highlighted patients’ concerns about being overheard or seen by others during online consultations [59]. Within the context of a trial, these issues need to be planned for and anticipated. Not only could they affect intervention fidelity and the validity of trial results [60, 61] they raise important questions around the potential risks associated with remote intervention delivery. For example, what happens if participants do not have the space to safely engage in a certain part of the intervention? What should trialists do if participants become distressed? Several papers in this review reported steps taken to manage these issues, for example, carrying out additional privacy and safety checks [41, 48]. However, it is unclear how effective these strategies employed by trialists were at mitigating these risks. Future work should identify how trialists can best plan for and mitigate the potential safety and privacy risks of delivering an intervention remotely into a participant’s home. The facilitators identified in this review provide a starting point for future investigation.

To address some of the safety and privacy concerns of remote intervention delivery, some trialists reported making changes to the eligibility criteria for intervention participation. For example, Johnson et al. [42] excluded people from participating remotely who had a high risk of spyware on their computer. Kashikar-Zuck et al. [51] describe how unless participants were in a private space, they were not allowed to participate in the remotely delivered group intervention. While these responses might reflect the limited time available to prepare for the safety challenges, it highlights the potential impact of remote intervention delivery on research inclusion for trials. The implications of remotely delivering an intervention using digital technologies on inclusivity have been widely discussed [62–64]. These trials, typically known as decentralised trials (DCTs), could make trials more accessible for individuals who are home bound [65, 66]. However, they could also introduce more severe health inequities for socially disadvantaged groups who experience greater challenges accessing digital health technologies [67, 68]. This review has highlighted several additional factors in the home setting that could influence inclusion in trials where the intervention is remotely delivered. For example, remote delivery for individuals living in crowded or unstable housing may pose logistical and practical challenges that hinder trial participation [69, 70].

This review demonstrated that remote delivery of a complex intervention can be achieved via several different modalities. The most frequently used mode of delivery was video call. Technological and connectivity challenges were commonly reported. To address these challenges, many trialists used additional resources and technological support to ensure participants were comfortable with the new setup. For example, equipment was provided for participants who needed it [41, 48] and equipment was checked to ensure functioning prior to intervention delivery [28, 31, 41]. Providing additional support to remotely deliver an intervention has also been identified as an unanticipated consideration of remote delivery in a recent qualitative study by Cooney et al. [10]. These responses likely incurred additional time and efficiency costs that are important considerations for the long-term viability of a trial intervention and its post-trial implementation [71, 72]. To support trialists to adapt an intervention for remote delivery, an understanding of the potential organisation and resource implications is required.

Many of the challenges trialists reported were specific to the modes of delivery used to deliver the intervention remotely. Connectivity issues were a particular challenge for delivery via video call, whereas adapting communication style was more of a consideration for intervention delivery via the telephone. Previous studies have also noted these mode of delivery specific challenges. Irvine et al. [73] emphasise the need for more explicit verbal cues during telephone interactions to demonstrate ‘active listening’, whereas technological challenges are frequently reported in video-based intervention delivery [74–76]. There is a growing interest in the ability to deliver an intervention remotely in a trial using digital technologies. However, there is limited guidance on how best to do this [18]. The results from this review demonstrate the value of developing guidance that is tailored to the type of mode of delivery being used. Different modes of delivery will require different practical and communication-related considerations.

Three reports in this review originally used a web-based mode of delivery to deliver all, or part of the intervention [24, 42, 46]. However, this did not make the intervention immediately suitable for remote delivery. Trialists reported challenges as a result of not being present with participants while they were set up with the intervention and engaged with it remotely [24, 46]. This finding suggests that the suitability of an intervention for remote delivery is not just related to whether it is delivered via a digital technology but also the degree to which it requires human facilitation and support. Evidence from other web-based interventions similarly indicates that effective participant engagement often depends on human support during onboarding and throughout the intervention [77]. Cooney et al. [10] highlight that there is not a dichotomous divide between in-person and digital delivery. They introduce the concept of a continuum of digitalisation to demonstrate the multiple points in between complete in-person delivery and fully automated delivery. Different considerations for adaptation are required depending on the degree of digitalisation and the degree of human support needed. This finding supports the need for clarity in the reporting of digitally delivered complex interventions [78]. It is important to report not just the mode of delivery used, but the degree of human support and facilitation required.

### Strengths and limitations

A key strength of this review is the use of the upper level of the Behaviour Change Intervention Ontology (BCIO) as a framework to organise the challenges and facilitators related to remote intervention delivery. The BCIO supported a more granular examination of where guidance is needed. For example, by distinguishing between different modes of delivery, it was possible to see the challenges that relate more specifically to the use of video call compared to the telephone. Another strength of this review is that the studies included are from a range of trials with different populations and trial designs. This supports the generalisability of results and learnings related to the remote delivery of a complex intervention within a trial.

The review had limitations. Most papers in this review came from trials conducted in the USA, which may limit generalisability of the results. Most studies in this review, however, reported challenges related to remote delivery in the home environment (e.g. interruptions and disruptions) and to intervention mode of delivery (e.g. technological difficulties with video conferencing software). It is reasonable to assume that these challenges have wide relevance and applicability.

The studies included in this review came from trialists who had successfully adapted an intervention for remote delivery. Therefore, insights from trialists who were unable to adapt their intervention were not included, and key, potentially insurmountable, challenges to intervention adaptation and remote delivery might be missed.

While this review has identified several ‘facilitators’ of both adaptation and remote intervention delivery, these are self-reported by trialists. It is unclear how effective they were in managing the challenges that were faced. The facilitators identified in this review can be used as a starting point for more in-depth investigation.

Finally, the literature from the COVID-19 period is still maturing. Therefore, a number of potentially eligible papers and relevant insights may be missed. The purpose of this scoping review, however, was not to definitively identify every lesson learned related to adaptation and remote delivery. Instead, it aimed to identify key characteristics in the data that can be used to inform more in-depth future research.

## Conclusion

This systematic scoping review identified a broad range of challenges that trialists faced as a result of remote intervention delivery. Little detail was provided about how trialists adapted their interventions for remote delivery. Key challenges of remote delivery in the home environment included privacy and safety concerns, which in some instances impacted trial participation. Technological and connectivity challenges were commonly reported with the use of video call. Suitability of an intervention for remote delivery depends not only on whether it is originally delivered via digital technology, but the extent to which it requires human facilitation and support. Further research is needed to explore how trialists can effectively prepare for and manage the challenges of remote delivery highlighted in this review. Any guidance that is developed to support the adaptation of an intervention for remote delivery within a trial should be specific to the mode of delivery used.

## Supplementary Information

Additional file 1: Final search strategy

Additional file 2: Data extraction table. Acronym list: BCIO, Behaviour Change Intervention Ontology; JBI, Joanna Briggs Institute; DCT, decentralised trial

## Data Availability

The protocol is available on the Open Science Framework. The extracted data is available upon request.
