# Supplementary material for: ‘Lessons learned’ from trialists who adapted a complex intervention for remote delivery within a trial as a result of the COVID-19 pandemic: a scoping review
Source: Trials. 2025 Nov 25;26:548. doi: 10.1186/s13063-025-09145-6 (PMC12648927; doi:10.1186/s13063-025-09145-6)
Supplement: Supplementary file 1 — Additional file 1: Final search strategy [file 13063_2025_9145_MOESM1_ESM.docx]

**Additional file 1: Search strategy**

**MEDLINE:**

((adapt* or transition* or modif* or adjust*) adj8 (research or study procedure* or protocol* or method* or intervention* or trial*) adj5 (web* or remot* or virtual*)).tw,kw.

**WEB OF SCIENCE**

TS=((web* or remot* or virtual*) NEAR/5 (research or "study procedure*" or protocol* or method* or intervention* or trial*) Near/8 (transition* or adapt* or adjust* or modif*))

**PsycINFO**

((adapt* or transition* or modif* or adjust*) adj8 (research or "study procedure*" or protocol* or method* or intervention* or trial*) adj5 (web* or remot* or virtual*)).ti,ab.

**EMBASE**

((adapt* OR transition* OR modif* OR adjust* ) ADJ8 (research OR "study procedure*" OR protocol* OR method* OR intervention* OR trial* ) ADJ5 (web* OR remot* OR virtual* ))

**COCHRANE:**

((adapt* OR transition* OR modif* OR adjust* ) NEAR/8 (research OR ("study" NEXT proceedure*) OR protocol* OR method* OR intervention* OR trial* ) NEAR/5 (web* OR remot* OR virtual* ))
