# Supplementary material for: ‘Lessons learned’ from trialists who adapted a complex intervention for remote delivery within a trial as a result of the COVID-19 pandemic: a scoping review
Source: Trials. 2025 Nov 25;26:548. doi: 10.1186/s13063-025-09145-6 (PMC12648927; doi:10.1186/s13063-025-09145-6)
Supplement: Supplementary file 2 — Additional file 2: Data extraction table. Acronym list: BCIO, Behaviour Change Intervention Ontology; JBI, Joanna Briggs Institute; DCT, decentralised trial [file 13063_2025_9145_MOESM2_ESM.docx]

**Additional file 2: Data extraction table**

| **Title and First author** | **Date of publication** | **Country** | **Aims** | **Study design** | **Study population** | **Study Intervention** | **Study comparator** | **Study outcome** |
| --- | --- | --- | --- | --- | --- | --- | --- | --- |
|  | *Year of publication* | *Where was the intervention carried out?* | *Aim of the study/paper* | *Study design (e.g. pilot, feasibility)* | *Who took part in the trial?* | *What was being tested?* | *What was the comparator?* | *What outcome was being recorded?* |

| **Mode of Delivery to deliver intervention content remotely** | **Challenges of process of adaptation** | **Facilitator of process of adaptation** | **Challenges of remote delivery** | **Facilitators of remote delivery** | **Additional insights/opportunities raised by trial teams** |
| --- | --- | --- | --- | --- | --- |
| *Which mode of delivery did triallists report using for remote delivery of the intervention (e.g. video call/ telephone)* | *What do trialists report as being a challenge during the adaptation process* | *What do trialists report as being a facilitator during the adaptation process* | *What do trialists report as being a challenge of remote delivery of the complex intervention* | *What do trialists report as facilitating/ supporting the remote delivery of the complex intervention* | *What additional insights do triallists discuss around their experience of being impacted by the COVID-19 pandemic, specifically related to adaptation and remote delivery.* |
